# Combined CT-guided radiofrequency ablation with systemic chemotherapy improves the survival for nasopharyngeal carcinoma with oligometastasis in liver: Propensity score matching analysis

**DOI:** 10.18632/oncotarget.10383

**Published:** 2016-07-02

**Authors:** Wang Li, Yutong Bai, Ming Wu, Lujun Shen, Feng Shi, Xuqi Sun, Caijin Lin, Boyang Chang, Changchuan Pan, Zhiwen Li, Peihong Wu

**Affiliations:** ^1^ Department of Medical Imaging and Interventional Radiology, Sun Yat-sen University Cancer Center, State Key Laboratory of Oncology in South China, Collaborative Innovation Center for Cancer Medicine, Guangzhou, Guangdong 51060, P. R. China; ^2^ Zhong Shan Medical School, Sun Yat-sen University, Guangzhou 510080, People's Republic of China; ^3^ Department of Medical Oncology, Sichuan Cancer Hospital and Institute, Second People's Hospital of Sichuan Province, Chengdu, Sichuan 610041, P. R. China

**Keywords:** Nasopharyngeal carcinoma, liver metastasis, radiofrequency ablation, palliative chemotherapy, prognosis

## Abstract

The aim of this study was to retrospectively compare the treatment efficacy of systemic chemotherapy combined with sequential CT-guided radiofrequency ablation (Chemo-RFA) to chemotherapy alone (Chemo-only) in the management of nasopharyngeal carcinoma (NPC) with liver metastasis. Between 2003 and 2011, 328 NPC patients diagnosed with liver metastasis at Sun Yat-sen University Cancer Center were enrolled. One-to-one matched pairs between Chemo-RFA group with the Chemo-only group were generated using propensity score matching. The associations of treatment modality with overall survival (OS) and progression-free survival (PFS) were determined by Cox regression. Of the patients enrolled, 37 patients (11.8 %) received combined treatment, 291 (82.2) received chemotherapy alone. The patients in Chemo-RFA group were more frequently classified as lower number (≤3) of liver metastatic lesions (P<0.001), had lower rates of bi-lobar liver metastasis (P<0.001) and extra-hepatic metastasis (P<0.001) than patients in Chemo-only group. After propensity score matching, 37 pairs of well-matched liver metastatic NPC patients were selected from different treatment groups. The adjusted hazard ratio in OS and PFS of the choice for Chemo-RFA approach to Chemo-only was 0.53 (95%CI, 0.30-0.93) and 0.60 (95%CI, 0.36-0.97), respectively. In conclusion, combined CT-guided RFA and chemotherapy approach offer the chance of improved survival for NPC patients with oligometastasis in liver, and should be considered if the ablation is technically feasible.

## BACKGROUND

Nasopharyngeal carcinoma (NPC) is an endemic head and neck epithelial malignancy in Southeast Asia [1, 2]. With the progress in radiation techniques and chemotherapy regimens, the local control of NPC has considerably improved and the predominant cause of treatment failure is distant metastasis [3]. Bone, lung, and liver are the most common metastasis sites of NPC, while liver metastasis confers the worst prognosis [4, 5]. Currently, the standard management of metastatic NPC is combined chemotherapy, which is rarely curative [6, 7].

Radiofrequency ablation (RFA), which employs high-energy radiofrequency waves to destroy abnormal tissue or tumors, is widely used in the treatment of hepatocellular carcinoma and various solid malignancies [8–10]. Its combination with chemotherapy is the standard therapeutic approach for colorectal liver metastases with potential to cure the disease [9, 11]. Recently, several reports in evaluating the survival benefit of RFA in liver metastatic NPC (LM-NPC) had been published. Pan et al. retrospectively analyzed the data of 17 LM-NPC patients receiving RFA treatment and found it was technically effective and could potentially lead to an improved survival [12]. Jin et al. analyzed a consecutive series of 134 LM-NPC patients and found RFA combined with chemotherapy could achieve higher local response and overall survival rates as compared with chemotherapy or RFA alone [13]. However, these studies still could not provide conclusive evidence due to the small sample size and the potential selection bias.

Therefore, in this study, we aim to compare the efficacy of chemo-RFA combination therapy with chemo-only therapy in LM-NPC patients through propensity score matching.

## RESULTS

### Baseline characteristics of chemo-RFA and chemo-only groups

Table 1 shows the patient characteristics for the two groups. Patients who underwent Chemo-RFA were more frequently classified as lower number (≤3) of liver metastatic lesions (P<0.001), had lower rates of bi-lobar liver metastasis (P<0.001) and extra-hepatic metastasis (P<0.001) than did patients who underwent chemotherapy only. There were no significant differences in the distribution of age, gender, UICC T stage, UICC N stage, KPS, metastatic onset, liver tumor size, cycles of chemotherapy between the two groups.

**Table 1:** Clinical characteristics of patients in the Combined therapy group and control group

Variable	Chemo-RFA Group (n=37)	Chemo-only Group (n=291)	P Value
**Age, years**			0.077
<45	23 (62.2)	136 (46.7)	
≥45	14 (37.8)	155 (53.3)	
**Gender**			0.402
Male	28 (75.7)	237 (81.4)	
Female	9 (24.3)	54 (18.6)	
**UICC T Stage**			0.891
T1-2	12 (32.4)	97 (33.6)	
T3-4	25 (67.6)	192 (66.4)	
**UICC N Stage**			0.987
N0-1	16 (43.2)	125 (43.1)	
N2-3	21 (56.8)	165 (56.9)	
**KPS**			0.074*
≥80	37 (100.0)	270 (92.8)	
<80	0 (0.0)	21 (7.2)	
**Metastatic onset**			0.905
Metachronous	22 (59.5)	176 (60.5)	
Synchronous	15 (40.5)	115 (39.5)	
**Liver Tumor Size**			
Mean±SD (cm)	2.81 ± 1.47	3.56 ± 3.38	0.185
Classification			0.114
<5cm	34 (91.9)	237 (81.4)	
≥5cm	3 (8.1)	54 (18.6)	
**Number of liver metastatases**			<0.001*
≤3	37 (100)	137 (47.1)	
>3	0 (0)	154 (52.9)	
**Extra-hepatic metastasis**			<0.001
Absent	24 (64.9)	67 (23.0)	
Present	13 (35.1)	224 (77.0)	
**Lobar location**			<0.001
Single	28 (75.7)	106 (36.4)	
Both	9 (24.3)	185 (63.6)	
**Cycles of Chemotherapy**			0.780
≤4	15 (40.5)	125 (43.0)	
>4	22 (59.5)	166 (57.0)	

Abbreviations: SD, standard deviation.

*Fisher's exact test.

### Technical success of RFA in chemo-RFA group

In the chemo-RFA group, a total of 50 of the 66 tumors were completely necrotized after the first session of ablation. Of 9 patients (9/37, 24.3%) in the chemo-RFA group, 3 lesions showed residual tumor and 13 tumors remained un-ablated after one-month follow-up. After the second and third session of ablation, 14 tumors were completely necrotized, as revealed by CT scan. Complete ablation of all liver metastases were achieved in 35 patients (35/37, 94.6%).

### OS and PFS

**Figure 1:**
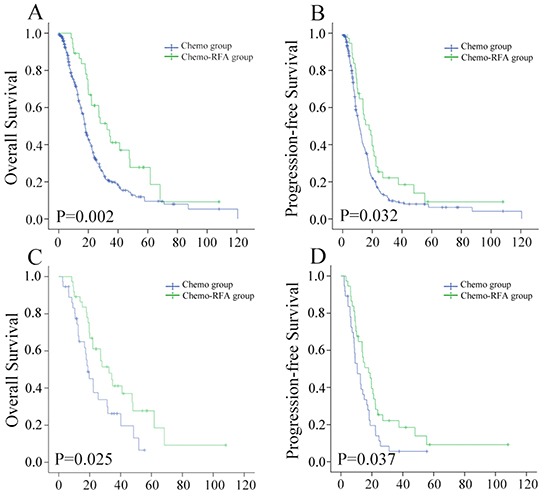
Kaplan–Meier curves of LM-NPC patients by different treatment modality in **A.** overall survival before matching; **B.** progression-free survival before matching; **C.** overall survival after matching; **D.** progression-free survival after matching.

The median follow-up time of all the patients was 15.4 months (range, 1-120 months). Among 37 patients received chemo-RFA, seven were still alive, 25 had died, and five, were lost to follow-up by the end of this study. Among the 291 patients received chemotherapy only, 19 were still alive, 201 had died, and 71 were lost to follow-up. The 1-, 3-, and 5-year OS rates were 89.0%, 41.3% and 29.5% in the chemo-RFA group, and 71.2%, 20.5% and 10.1% in the chemo-only group, respectively. OS rates in the chemo-RFA group significantly exceeded those in the chemo-only group (P = .002; Figure 1A). In all patients, treatment modality (chemo-RFA/chemo-only), UICC T stage (T3-4/T1-2), KPS (<80/≥80), liver tumor size (≥5cm/<5cm), number of liver metastases (>3/≤3), Extra-hepatic metastasis (present/absent), lobar location (both/single), and cycles of chemotherapy (>4/≤4) were significantly associated with the OS in univariate analysis (Table 2). Multivariate analysis showed UICC T Stage (P=0.007), Number of liver metastases (P=0.001), Extra-hepatic metastasis (P=0.016), and cycles of chemotherapy (P<0.001) were independent predictors, while the treatment of chemo-RFA as opposed to chemo-only, was not an independent risk factor (P=0.087; Table 2).

**Table 2:** Univariate and multivariate analysis of OS in the entire cohort of NPC patients with liver metastasis

Variable	No. of Cases	Univariate Analysis		Multivariate Analysis	
		HR (95% CI)	P value	HR (95% CI)	P value
Modality (Chemo-RFA vs Chemo-only)	37/291	0.53 (0.35-0.80)	0.001	0.67 (0.43-1.06)	0.087
Age (≥45 years vs <45 years)	169/159	0.95 (0.73-1.23)	0.685	-	-
Gender (Female vs Male)	63/265	0.77 (0.55-1.10)	0.150	-	-
UICC T Stage (T3-4 vs T1-2)	217/111	1.35 (1.02-1.78)	0.033	1.47 (1.11-1.95)	0.007
UICC N Stage (N2-3 vs N0-1)	186/142	1.02 (0.78-1.32)	0.912	-	-
KPS (<80 vs ≥80)	21/307	1.90 (1.10-3.28)	0.021	1.69 (0.97-2.96)	0.064
Metastatic Onset (Synchronous vs Metachronous)	130/198	0.94 (0.72-1.23)	0.672	-	-
Liver Tumor Size (≥5cm vs <5cm)	57/271	1.56 (1.12-2.18)	0.008	-	-
Number of liver metastatases (>3 vs ≤3)	154/174	1.57 (1.20-2.04)	0.001	1.63 (1.23-2.17)	0.001
Extra-hepatic metastasis (Present vs Absent)	237/91	1.65 (1.22-2.23)	0.001	1.51 (1.08-2.10)	0.016
Lobar Location (Both vs Single)	194/134	1.53 (1.17-2.01)	0.002	-	-
Cycles of Chemotherapy (>4 vs ≤4)	188/140	0.48 (0.37-0.63)	<0.001	0.42 (0.32-0.55)	<0.001

Abbreviations: HR, hazard ratio; KPS, Karnofsky performance score.

Progression of disease was observed in 31 patients (83.8%) who underwent chemo-RFA, and 232 patients who underwent chemo-only (79.7%) during follow-up. The 1-, 3-, and 5-year OS rates were 64.4%, 22.0% and 8.4% in the chemo-RFA group, and 50.1%, 9.1% and 6.6% in the chemo-only group, respectively. Patients in chemo-RFA group had significantly higher PFS rates as compared with those in chemo-only group (P=0.032; Figure 1B). In all patients, treatment modality, KPS, liver tumor size, number of liver metastases, extra-hepatic metastasis, lobar location, and cycles of chemotherapy were significantly associated with the PFS in univariate analysis (Table 3). Multivariate analysis showed KPS (P=0.048), number of liver metastases (P<0.001), and cycles of chemotherapy (P=0.005) were independent predictors, while the treatment of chemo-RFA as opposed to chemo-only was not an independent risk factor for PFS (Table 3).

**Table 3:** Univariate and multivariate analysis of PFS in the entire cohort of NPC patients with liver metastasis

Variable	No. of Cases	Univariate Analysis		Multivariate Analysis	
		HR (95% CI)	P value	HR (95% CI)	P value
Modality (Chemo-RFA vs Chemo-only)	37/291	0.67 (0.46-0.97)	0.034	-	-
Age (≥45 years vs <45 years)	169/159	0.96 (0.75-1.23)	0.743	-	-
Gender (Female vs Male)	63/265	0.95 (0.70-1.30)	0.755	-	-
UICC T Stage (T3-4 vs T1-2)	217/111	1.16 (0.90-1.50)	0.262	-	-
UICC N Stage (N2-3 vs N0-1)	186/142	1.06 (0.83-1.36)	0.912	-	-
KPS (<80 vs ≥80)	21/307	1.70 (1.02-2.84)	0.041	1.68 (1.00-2.80)	0.048
Metastatic Onset (Synchronous vs Metachronous)	130/198	0.88 (0.68-1.12)	0.672	-	-
Liver Tumor Size (≥5cm vs <5cm)	57/271	1.41 (1.04-1.93)	0.030	-	-
Number of liver metastatases (>3 vs ≤3)	154/174	1.52 (1.19-1.95)	0.001	1.62 (1.26-2.07)	<0.001
Extra-hepatic metastasis (Present vs Absent)	237/91	1.23 (0.94-1.62)	0.018	-	-
Lobar Location (Both vs Single)	194/134	1.35 (1.05-1.73)	0.019	-	-
Cycles of Chemotherapy (>4 vs ≤4)	188/140	0.73 (0.57-0.94)	0.015	0.70 (0.54-0.90)	0.005

### One-to-one propensity score analysis

**Figure 2:**
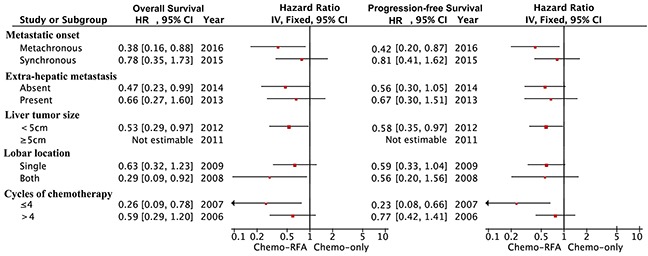
Forest plot of hazard ratios showing the impact of treatment modality on OS and PFS in stratified analysis

A total of 37 patients from each group were matched by applying one-to-one propensity score matching, with confounding factors well matched (Table 4). The median OS were 32.5 months in chemo-RFA group and 18.8 months in the chemo-only group, respectively. The 1-, 3-, and 5-year OS rates were 89.0%, 41.3% and 29.5% in the chemo-RFA group, and 74.3%, 25.3% and 3.9% in the chemo-only group, respectively. The 1-, 3-, and 5-year PFS rates were 64.4%, 22.0% and 8.4% in the chemo-RFA group, and 47.9%, 5.6% and 5.6% in the chemo-only group. Patients in the chemo-RFA group had significantly higher OS (P=0.025) and PFS rates (P=0.037) when compared with patients in chemo-only group (Figure 1C, 1D). The adjusted hazard ratio in OS and PFS of the choice for Chemo-RFA approach to Chemo-only was 0.53 (95%CI, 0.30-0.93) and 0.60 (95%CI, 0.36-0.97), respectively. Further stratified analysis by known prognostic factors was further explored to the treatment impact of chemo-RFA as opposed to chemo-only (Table 5; Figure 2).

**Table 4:** Clinical characteristics of patients in the combined therapy and control groups by propensity analysis with One-to-one Nearest-Neighbor Caliper Matching Method

Variable	Chemo-RFA Group (n=37)	Chemo-only Group (n=37)	P Value
**Age, years**			0.809
<45	23 (62.2)	24 (64.9)	
≥45	14 (37.8)	13 (35.1)	
**Gender**			1.000
Male	28 (75.7)	28 (75.7)	
Female	9 (24.3)	9 (24.3)	
**UICC T Stage**			0.338
T1-2	12 (32.4)	16 (43.2)	
T3-4	25 (67.6)	21 (56.8)	
**UICC N Stage**			0.636
N0-1	16 (43.2)	14 (37.8)	
N2-3	21 (56.8)	23 (62.2)	
**KPS**			-
≥80	37 (100.0)	37 (100.0)	
<80	0 (0.0)	0 (0.0)	
**Metastatic onset**			0.063
Metachronous	22 (59.5)	14 (37.8)	
Synchronous	15 (40.5)	23 (62.2)	
**Liver Tumor Size**			
Mean±SD (cm)	2.81 ± 1.47	3.03 ± 1.95	0.587
Classification			1.000
<5cm	34 (91.9)	34 (91.9)	
≥5cm	3 (8.1)	3 (8.1)	
**Number of liver metastatases**			1.000
≤3	37 (100.0)	37 (100.0)	
>3	0 (0.0)	0 (0.0)	
**Extra-hepatic metastasis**			1.000
Absent	24 (64.9)	24 (64.9)	
Present	13 (35.1)	13 (35.1)	
**Lobar location**			0.782
Single	28 (75.7)	29 (78.4)	
Both	9 (24.3)	8 (21.6)	
**Cycles of Chemotherapy**			0.330
≤4	15 (40.5)	11 (29.7)	
>4	22 (59.5)	26 (70.3)	

**Table 5:** Hazard ratios for OS and PFS by treatment modality stratified by covariates in propensity score-matched pairs

Stratification Covariates	Patients, n	Chemo-RFA therapy/Chemo-only therapy			
		OSHR	95%CI	PFSHR	95%CI
**Metastatic Onset**					
Metachronous	36	**0.38**	0.16-0.88	**0.42**	0.20-0.88
Synchronous	38	0.75	0.34-1.67	0.81	0.41-1.62
**Extra-hepatic metastasis**					
Absent	48	**0.34**	0.15-0.79	0.56	0.30-1.05
Present	26	0.48	0.16-1.43	0.67	0.30-1.51
**Liver Tumor Size**					
<5cm	68	0.45	0.24-0.84	0.51	0.29-0.89
≥5cm	6	-	-	-	-
**Lobar location**					
Single	57	0.63	0.33-1.23	0.59	0.33-1.04
Both	17	0.29	0.09-0.92	0.56	0.20-1.56
**Cycles of Chemotherapy**					
≤4	26	**0.26**	0.09-0.78	**0.24**	0.08-0.66
>4	48	0.59	0.29-1.20	0.77	0.42-1.41

The protective impact of chemo-RFA therapy was not significant among patients with synchronous metastasis, or extra-hepatic metastasis, or those receiving more than four cycles of chemotherapy.

## DISCUSSION

In this study, we found that combined CT-guided RFA and systemic chemotherapy could improve OS and PFS for NPC patients with liver metastasis as compared with chemotherapy alone. This effect was more pronounced among patients with metachronous metastasis, no extra-hepatic metastasis and received less (≤4) cycles of chemotherapy. Our results of propensity score matching provided substantial evidence in support of the combined Chemo-RFA therapy in the management of liver metastasis of NPC in the current era.

Distant metastasis is the major cause of treatment failure and death in NPC [2]. In recent years, there is growing evidence that the prognosis of patients with metastatic NPC varied greatly [5, 14, 15]; lung-only metastasis seemed to be correlated with improved survival while liver metastasis conferred the worst prognosis [16]. For isolated location metastasis, the number of metastatic lesions and location of the lesion were also important implication [17, 18]. Pan et al. retrospectively review the data of 305 NPC patients with liver metastasis and their results showed better survival was independently predicted by having one to three (vs. more than three) metastatic lesions (HR, 0.52; 95% CI 0.33-0.82) and unilobular (vs. bilobular) lesions (HR, 0.35; 95% CI 0.22-0.57)[19]. All these evidences in metastatic NPC gave us support in exploration of better therapeutic approach for potentially curable disease.

Several studies published in the past demonstrated that ablative therapies, including RFA and microwave ablation, could offer as safe and effective treatment alternatives for local tumor control in LM-NPC [12, 20]. A recently published study involving a large consecutive series showed RFA combined with chemotherapy could achieve higher local response and overall survival rates as compared with chemotherapy or RFA alone [13]. However, in their study, the metastatic onset and cycles of chemotherapy received had not been included in an analysis, and a marginally significant difference in the distribution of the number of the liver metastatic lesion was observed between different treatment subgroups; all these factors might cause confounding effects. In our study, a significantly different distribution of number of liver metastases (≤3/>3), lobar location (single/both) and extra-hepatic metastasis (absent/present) were considered between Chemo-RFA group and Chemo-only group. And the impact of Chemo-RFA treatment on OS and PFS were not found significant in multivariate analysis, indicating potential interaction effect between covariates and treatment modality may exist. To thoroughly investigated the treatment effect of Chemo-RFA combination therapy among liver metastatic NPC, we conducted a propensity score matching analysis, with 11 covariates included. Our results suggested chemo-RFA combination therapy could achieve better treatment outcome as compared to chemotherapy alone in the LM-NPC.

In stratified analysis, no significant survival benefit of chemo-RFA therapy was obtained from patients with synchronous metastasis, or extra-hepatic metastasis, or received more than four cycles of chemotherapy, suggesting that the patient selection for combined treatment should be personalized [21, 22]. Combined chemo-RFA therapy for LM-NPC patients with synchronous metastasis or extra-hepatic metastasis should be conducted after sufficient counseling and discussion in case of excessive medical treatment; while for patients can't tolerate the toxicity of chemotherapy or unwilling to receive more than four cycles of chemotherapy, RFA treatment is recommended. It needed to be noted that in the Chemo-RFA group, 8.1% of the patients had liver metastasis with more than 5 cm in diameter, and 5.4% didn't achieve the technical success of complete ablation of all the identified metastatic lesions, which was a minuscule proportion. Therefore, applying the results of our study in these group of patients should be cautious.

This study has several limitations. First, it is a retrospective study. Second, the patients enrolled in our match-pair analysis is a very heterogeneous group, including patients with and without extra-hepatic metastasis; the actual impact of combined Chemo-RFA treatment in specific patient group warrants further investigation. Finally, the modes of chemotherapy applied varied, which might have a confounding effect. For these reasons, a multi-institutional clinical trial is needed in the future.

## MATERIALS AND METHODS

### Patients

**Figure 3:**
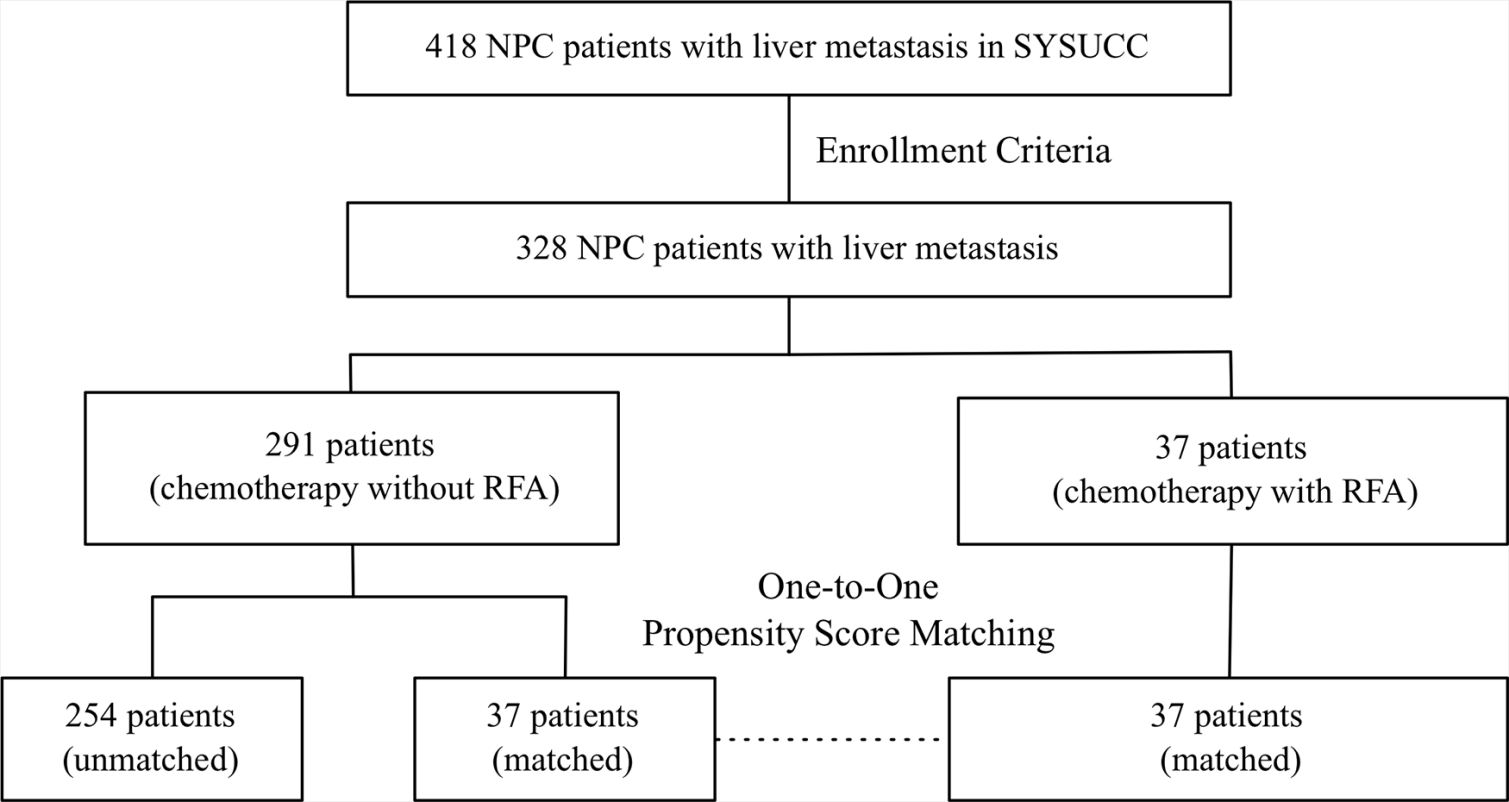
Flowchart of study design A total of 328 patients met the enrollment criteria. Thirty-seven 1:1 match-pairs were generated using propensity score matching.

The medical records of 418 NPC patients diagnosed with liver metastasis in Sun Yat-sen University Cancer Center (SYSUCC) from Jan, 2003 to December, 2011 were reviewed. This study was approved by SYSUCC Hospital Ethics Committee, which waived the need for written informed consent because of the retrospective nature of the study. The inclusion criteria included: (1) histologically confirmed NPC; (2) diagnosis of liver metastasis at the time of initial staging or developed liver metastasis as the first recurrence site during follow-up; (3) received at least one cycle of systemic chemotherapy as first-line treatment after metastasis. The exclusion criteria included: (1) refuse to receive treatment in our hospital (n=6); (2) incomplete presence of pretreatment evaluation including computed tomography (CT) or magnetic resonance imaging (MRI) scans of the head and neck regions, radiographs/CT scans of the chest, sonography/CT scans of the abdomen, and whole-body bone scan (n=24); (3) using other treatment modality as first line treatment after liver metastasis except for chemotherapy and CT-guided RFA (n=60). A total of 328 patients were enrolled (Figure 3). The histology in 96.0% of the patients was non-keratinizing or undifferentiated carcinoma (World Health Organization [WHO] type 2 or 3 histology) [23]. The median duration of follow-up for the population was 16 months (range, 1-120 months).

### Treatment

Treatment modalities were defined as the first-line treatment towards the metastatic liver lesions from the time of diagnosis of liver metastasis to the time of disease progression or mortality. Chemotherapy regimens in the study were all cisplatinum-based, with cisplatin in combination with one or two of the following drugs: fluorouracil (5-FU), cyclophosphamide, vincristine, paclitaxel, and bleomycin, for 4-6 cycles. A change of chemotherapy regimen would be conducted if the metastatic disease progressed under the primary treatment. Treatment discontinuation occurred as a request by patients or for unacceptable drug toxicities.

RFA was performed percutaneously under moderate sedation and local anaesthesia. Sedation was induced with intravenous administration of midazolam (2.5∼5.0 mg; Roche, Basel, Switzerland) and propofol injection (50∼100 mg; AstraZeneca, Italy). CT was used to guide placement of the RF needle into the lesions to be treated by RFA. Single/clustered needle electrode(s) with a 2, 3 or 4 cm exposed tip were used. Power settings and exposure times were selected according to the standard recommendations provided by the manufacturers of the equipment used and the preferences of the individual operators, depending on our previous ablation experience, with the aim to completely necrotize the tumour. We used 1∼3 ablation sites per lesion to ensure destruction of the entire target tumour and approximately 1cm of surrounding tissue. Technical success was defined as no focal nodular enhancement by dynamic CT scan obtained at least 3 months after treatment. Of the 37 patients who underwent chemotherapy concurrent with RFA, 13 patients received RFA followed by chemotherapy and 24 patients received chemotherapy followed by RFA. The time interval between the two modalities was usually 3-4 weeks.

### Follow-up and end point

Patients were evaluated for response every two cycles during systemic chemotherapy and then every three months until death, based on computed tomography (CT) or magnetic resonance imaging (MRI). The primary endpoint was overall survival (OS), which was defined as the time from the treatment of distant metastasis to death by any causes. The secondary endpoint was progression-free survival (PFS), which was defined as the time from the treatment of distant metastasis to disease progression or death by any causes.

### Statistical analysis

Pearson χ test was used to compare categorical variables between groups, respectively. Rates of OS and PFS between the combined treatment group and chemotherapy only group were estimated by means of the Kaplan-Meier method and were compared between different subgroups with the use of the log-rank test. The independent prognostic factors for survival were determined by, multivariate Cox regression model in backward conditioned method was utilized, with the covariates including treatment modality, age, gender, UICC T stage, UICC N stage, KPS, metastatic onset, liver tumor size, number of liver metastases, extra-hepatic metastasis, lobar location and cycles of chemotherapy.

To minimize the effect of potential confounders on selection bias, propensity score matching analysis was performed to adjust for potential biases by selecting factors related to combined treatment. The selected variables entered into the propensity model included age, gender, UICC T stage, UICC N stage, KPS, metastatic onset, liver tumor size, number of liver metastases, extra-hepatic metastasis, lobar location and cycles of chemotherapy. One-to-one matching between the groups was accomplished by using the nearest-neighbor matching method, with a caliper of 0.25. The adjusted comparisons by propensity scores were based on data from 37 patients per treatment arm. After adjustment for these factors, OS and DFS rates were recalculated for the two groups. In addition, adjusted Cox regression model incorporating independent prognostic factors and the treatment modality (combined vs chemotherapy) were conduced in the stratified analysis to determine the prognostic impact of combined treatment among subgroups. All analyses were done using SPSS 20.0 or R 3.1.2 (The R Foundation for Statistical Computing, 2014).

## CONCLUSION

Combined CT-guided RFA and chemotherapy approach offer the chance of improved survival for NPC patients with liver metastasis and is recommended for patients with the metachronous liver metastasis, no extra-hepatic metastasis and the maximal diameter of liver lesion less than 5cm.
